# Cyclic Nucleotide-Gated Ion Channel 6 Mediates Thermotolerance in *Arabidopsis* Seedlings by Regulating Hydrogen Peroxide Production *via* Cytosolic Calcium Ions

**DOI:** 10.3389/fpls.2021.708672

**Published:** 2021-07-14

**Authors:** Wenxu Wang, Jiaojiao Zhang, Lijuan Ai, Dan Wu, Bing Li, Lingang Zhang, Liqun Zhao

**Affiliations:** ^1^Hebei Key Laboratory of Molecular and Cellular Biology, Key Laboratory of Molecular and Cellular Biology of the Ministry of Education, Hebei Collaboration Innovation Center for Cell Signaling, College of Life Sciences, Hebei Normal University, Shijiazhuang, China; ^2^College of Life Sciences, Inner Mongolia University, Hohhot, China

**Keywords:** heat shock, heat shock (stress) proteins, hydrogen peroxide, *Arabidopsis*, calcium ion

## Abstract

We previously reported the involvement of cyclic nucleotide-gated ion channel 6 (CNGC6) and hydrogen peroxide (H_2_O_2_) in plant responses to heat shock (HS). To demonstrate their relationship with plant thermotolerance, we assessed the effect of HS on several groups of *Arabidopsis* (*Arabidopsis thaliana*) seedlings: wild-type, *cngc6* mutant, and its complementation line. Under exposure to HS, the level of H_2_O_2_ was lower in the *cngc6* mutant seedlings than in the wild-type (WT) seedlings but obviously increased in the complementation line. The treatment of *Arabidopsis* seeds with calcium ions (Ca^2+^) increased the H_2_O_2_ levels in the seedlings under HS treatment, whereas treatment with a Ca^2+^ chelator (EGTA) inhibited it, indicating that CNGC6 may stimulate the accumulation of H_2_O_2_ in a manner dependent on an increase in cytosolic Ca^2+^ ([Ca^2+^]_cyt_). This point was verified by phenotypic observations and thermotolerance testing with transgenic plants overexpressing *AtRbohB* and *AtRbohD* (two genes involved in HS-responsive H_2_O_2_ production), respectively, in a *cngc6* background. Real-time reverse transcription-polymerase chain reactions and Western blotting suggested that CNGC6 enhanced the gene transcription of HS factors (HSFs) and the accumulation of HS proteins (HSPs) *via* H_2_O_2_. These upon results indicate that H_2_O_2_ acts downstream of CNGC6 in the HS signaling pathway, increasing our understanding of the initiation of plants responses to high temperatures.

## Introduction

Global warming is a serious environmental threat, and is an important limiting factor for normal plant growth and development. As fixed organisms, plants cannot escape from high temperature, but they have evolved methods and morphological variations to escape from its negative effects. As a countermeasure to heat shock (HS), plants can synthesize a series of HS proteins (HSPs) in the responses of cell to HS conditions. They act as molecular chaperones, ubiquitin, and certain proteases to counteract protein denaturation, aggregation, and degradation, which protect the plant cells from heat-damage (Lawas et al., 2018). Thus, the synthesis of HSP is especially important for plant survival under HS conditions. In eukaryotes, HSP induction is dependent on HS factors (HSFs), which act as transcription factors to be bound in HS elements in the promoter regions of HSP genes (Akerfelt et al., 2010).

Several reactive oxygen species (ROS) are constantly generated as by-products of aerobic metabolism at multiple locations in plant cells, including the photosynthetic electron transport chain in chloroplasts, NADPH oxidase in the plasma membrane (PM), and peroxidase in the cell wall (Gechev and Hille, 2005). They are always greatly toxic and swiftly detoxified by different cellular enzymatic and nonenzymatic mechanisms. In other situation, plants purposefully release ROS as signal molecules to initial various biological processes including stress defense, programmed cell death, and stomatal behavior. Hydrogen peroxide (H_2_O_2_), as the major and most stable type of ROS, plays a key role in resistance reactions in plant cells, and it primarily originates from PM NADPH oxidase. In *Arabidopsis*, NADPH oxidase is encoded by 10 genes, from *AtRbohA* to *AtRbohJ*, which have distinct and shared biological features (Macpherson et al., 2008).

For example, H_2_O_2_ generated from AtRbohD and AtRbohF acts as a signaling molecule in ABA-induced stomatal closure and is crucial for jasmonic acid-induced expression of genes controlled by the MYC2 transcription factor (Maruta et al., 2011; Iwai et al., 2019), but regulates lateral root development negatively by altering the localization of superoxide in primary roots of *Arabidopsis* (Li et al., 2015). Under Cd stress, the differential regulation of H_2_O_2_ metabolism, redox homeostasis, and nutrient balance by AtRbohC, AtRbohD, and AtRbohF is of potential interest for biotechnology applications for the phytoremediation of polluted soils (Gupta et al., 2017). AtRbohF is considered a key modulator of defense-associated metabolism and a crucial factor in the interplay between intracellular oxidative stress and pathogenesis responses in *Arabidopsis* (Chaouch et al., 2012). In addition, the level of H_2_O_2_ has been reported to increase following exposure to high temperatures, resulting in elevated HSF activation and HSP accumulation (Banti et al., 2010), whereas peroxide scavengers and inhibitors of H_2_O_2_ generation inhibited HSP expression in HS-exposed plants (Königshofer et al., 2008), implicating the involvement of H_2_O_2_ in the HS signaling pathway. Mutations in *AtRbohB* and *AtRbohD*, two isoforms of NADPH oxidase which contribute to H_2_O_2_ production, were reported to show weaker defects under HS (Larkindale et al., 2005). Our work further indicated that AtRbohB and AtRbohD-dependent H_2_O_2_ production acts upstream of nitric oxide (NO) in the HS signaling pathway, involving variations in HSF DNA-binding activity and HSP expression (Wang et al., 2014).

Calcium ions (Ca^2+^) mobilization is a core issue in various plant signaling pathways. Cyclic nucleotide-gated ion channels (CNGCs) are nonselective cation channels and the main entrances for Ca^2+^ influxes into cells (Jha et al., 2016). In *Arabidopsis* genome, there are 20 expressed CNGC genes, having both different and shared biological activities (Talke et al., 2003). For example, cyclic nucleotide-gated ion channel 6 (CNGC6), CNGC9, and CNGC14 fulfill part of redundant functions to generate and maintain tip focused Ca^2+^ oscillations, which are essential for proper root hair growth and polarity (Brost et al., 2019). CNGC2 and CNGC4-mediated Ca^2+^ entry is suggested to provide a vital link between the pattern-recognition receptor complex and Ca^2+^-dependent immunity programs in PAMP-triggered immunity signal pathways in plants (Tian et al., 2019). The pollen-tube-specific CNGC7, CNGC8, and CNGC18 together with calmodulin (CaM) constitute a molecular switch that control the open or close of the calcium channel depending on cellular Ca^2+^ levels (Pan et al., 2019). CNGC9 is reported to mediate the elevation of cytosolic Ca^2+^ ([Ca^2+^]_cyt_) to resist disease in rice (Wang et al., 2019). CNGCs are also believed to mediate Ca^2+^ signals in the HS pathway. We reported that CNGC6, a heat-responsive PM Ca^2+^-permeable channel, is associated with the expression of HSP genes and the acquisition of thermotolerance in *Arabidopsis* (Gao et al., 2012). CNGC6 *via* Ca^2+^ signaling initiates plant resistant reactions to heat stress, but its precise regulatory mechanisms remain obscure. Further investigations into HS signaling will enrich our understanding of the initial heat stress signaling processes.

Calcium ions and H_2_O_2_ are well known as two universal intracellular secondary messengers. Studies of plants have shown a close relationship between their individual pathways; however, there is controversy regarding which one is upstream of the other. Lots of studies implicate a specific role of H_2_O_2_ in regulating Ca^2+^ signaling. For example, H_2_O_2_ production regulates the elevation of [Ca^2+^]_cyt_ in ABA signaling pathways in *Arabidopsis* guard cells (Jiang et al., 2013; Islam et al., 2019). On the contrary, some studies have pointed to the role of Ca^2+^ in influencing H_2_O_2_ signaling. For example, extracellular Ca^2+^ through H_2_O_2_ alleviates NaCl-induced stomatal openings in *Vicia* guard cells (Zhao et al., 2011). Also, crosstalk between Ca^2+^ signaling and H_2_O_2_ is required for some signaling networks, for example, their co-operation in the process of heavy metal stress resistance (Nazir et al., 2020). The relationship between Ca^2+^ and H_2_O_2_ is not yet fully understood in plants exposed to HS conditions.

In this investigation, we used the model plant *Arabidopsis* to explore the relationship between H_2_O_2_ and the Ca^2+^-permeable channel CNGC6 under heat stress conditions. Our results demonstrate the involvement of H_2_O_2_ in CNGC6 signaling as a downstream factor in the HS signaling pathway, by stimulating *Hsf* transcription and HSP accumulation.

## Materials and Methods

### Plant Materials and Growth Conditions

The wild-type (WT) and mutant *Arabidopsis* were Col-0 ecotype. *atrbohB* and *atrbohD* mutant seeds were obtained from Dr. Miguel Angel Torress (University of North Carolina). The triple mutant *cngc6/rbohB/D* was obtained by crossing, while the transgenic lines *cngc6*/*35S*::*RbohB-1*, *cngc6*/*35S*::*RbohB-2*, *cngc6*/*35S*::*RbohD-1*, and *cngc6*/*35S*::*RbohD-2* were obtained using the floral dip method.

The *Arabidopsis* seeds were surface sterilized in 2% (v/v) sodium hypochlorite for 1 min and then washed thoroughly with water. The sterilized seeds were placed on Murashige and Skoog (MS) medium containing 3% sucrose and 0.7% agar and kept at 4°C in the dark for 3 days. The plants were then transferred to a growth chamber set at 22°C and 120 μmol m^−2^s^−1^ on a 16-h daily light period.

For chemical treatment, 2 ml of H_2_O_2_ at various concentrations (0, 25, 50, 100, and 200 μM; Sigma-Aldrich, St. Louis, MO) were sprinkled onto the leaf surfaces of 8-day-old seedlings after filter sterilization. Sterilized water was used as a substitute for the control of seedlings. After 8 h of pre-treatment, the seedlings were subjected to HS conditions (Wang et al., 2014). In addition, 5 mM CaCl_2_ or 2 mM EGTA (these reagents were prepared with sterilized water) was used to pre-treat the WT, *cngc6*, and COM12 seeds for 30 min before their being placed on MS medium in the fluorescence experiment, with sterilized water as the control.

### Thermotolerance Testing

About 8-day-old seedlings, grown at 22°C, were incubated in sterilized 5 mM CaCl_2_ at 37°C for 30 min, returned to 22°C for 2 h, then challenged at 45°C for 100 min, and then returned to 22°C for 5 days of recovery (Lewis et al., 2016). The seedlings that were still green and continuing to produce new leaves were registered as survivors. For Western blotting, 10-day-old seedlings were kept at 37°C for 2 h and collected for the analyses of HSP accumulation. All the experiments were repeated at least three times, and there were three independent biological replicates in each repeat (Peng et al., 2019).

### Fluorescence Microscopy

Hydrogen peroxide was visualized using the specific fluorescent probe 5-(and-6)-chloromethyl-29,79-dichlorodihydrofluorescein diacetate (CM-H_2_DCFDA; Invitrogen) as described previously (Wang et al., 2010) with some modifications. Wild-type and mutant seedlings were incubated in 1 ml of liquid MS medium (pH 5.8) with 10 μM CM-H_2_DCFDA for 20 min. Thereafter, the roots were washed three times for 15 min each in liquid MS medium prior to visualization with a fluorescence microscope (Eclipse TE 200, Nikon, Tokyo, Japan). The signal intensities were calculated using MetaMorph (Molecular Devices, Sunnyvale, CA).

### Vector Construction and the Generation of Transgenic Plants

To generate the *35S:6×Myc-RbohB* construct, the full-length *RbohB* coding sequence was amplified using the primers 5'-CGGGATC-CATGCGGGAGGAAGAAATG-3' and 5'-TCCACAAGGAAAATTTCTAGCTGCAGTT-3'. To generate the *35S:6×Myc-RbohD* construct, the full-length *RbohD* coding sequence was amplified with the primers 5'-CGGGATCCATGAAAATGAGACGAGGCAA-3' and 5'-CCACAAAGAGAACTTCTAGCTGCAGTT-3'. The products were cloned in the *pCAMBIA1307-6×Myc* vectors using the BamHI and PstI sites.

The transformation of the constructs into *Arabidopsis* (*cngc6*) was performed according to the floral dip method (Clough and Bent, 1998) with *Agrobacterium tumefaciens* (strain GV3101). Transformants were screened on plates containing 15 mg l^−1^ of Basta. Homozygous T3 transgenic lines were selected for further analysis.

### RT-qPCR Analysis

Total RNA (500 ng) was isolated from 10-day-old seedlings at 37°C for 1 h with a PrimeScript RT Reagent Kit (Takara Bio Inc., Otsu, Japan) for first-stand cDNA synthesis, as per the manufacturer’s instructions. The program was as follows: initial polymerase activation for 10 s at 95°C followed by 40 cycles of 95°C for 5 s and 60°C for 31 s. The reactions were performed using an ABI Prism 7,000 sequence detection system (Applied Biosystems, Foster City, CA) with SYBR Premix Ex Taq (Takara Bio Inc.). Primer pairs were designed using Primer Express (Applied Biosystems). Detailed primer sequences are shown in Supplementary Table 1.

### Western Blot Analysis

About 10-day-old seedlings were kept at 37°C for 2 h and then ground in liquid nitrogen. Total protein was extracted with an extraction buffer (10 mM HEPES, pH 7.9, containing 0.4 M NaCl, 0.5 mM dithiothreitol, 0.1 mM EDTA, 5% glycerol, and 0.5 mM phenylmethanesulfonyl fluoride), and the extracts were purified by centrifugation at 14,000 × *g* for 20 min at 4°C. The supernatants were transferred to fresh tubes, and the protein content was measured according to the description of Bradford (1976). Total proteins (50 μg) were analyzed by Western blotting, as described previously (Wang et al., 2014).

### Preparation of Protoplasts and Electrophysiology Analysis

Protoplasts were isolated as described previously (Demidchik and Tester, 2002) from 1 cm long of root tips of *Arabidopsis* seedlings cultivated vertically at 22°C for 8 days. Whole-cell voltage patch-clamping was carried out as described previously (Gao et al., 2012; Peng et al., 2019; Niu et al., 2020) with minor modification. Patch-clamp pipettes were pulled on a vertical electrode puller. The electrode was filled with pipette solution [0.5 mM CaCl_2_, 2 mM Mg-ATP, 0.5 mM Tris-ATP, 4 mM Ca(OH)_2_, 10 mM EGTA and 15 mM HEPES/Tris, pH 7.2, adjusted to an osmolality of 300 mOsm/kg with sorbitol; free Ca^2+^ concentration 100 nM]. The basal external solution comprised 10 mM CaCl_2_ and 5 mm MES/Tris, pH 5.8, adjusted to an osmolality of 300 mOsm/Kg with sorbitol. The resistance of the electrode in the bath solution was approximately 20 MΩ. Seal resistances were up to 2 GΩ. After holding the whole-cell high seal resistances for 20 min, currents were recorded and data were sampled at 1 kHz and filtered at 200 Hz. Membrane potentials were corrected for liquid junction potentials and series resistance. An Axon 200B amplifier controlled by pCLAMP 9.0 software (Molecular Devices) was used to record the current signal. Basal currents were recorded at room temperature (20–22°C). HS treatment (37 ± 1°C) was performed using continuous bath perfusion.

## Results

### Effects of HS on H_2_O_2_ Production in the Wild-Type, *cngc6*, and a Complemented Line COM12 Seedlings

In this work, we presented evidence for the involvement of H_2_O_2_ in Ca^2+^ signaling in plant thermotolerance. CNGC6, activated by HS and mediated Ca^2+^ influxes, functioned as a signal in the induction of H_2_O_2_ generation to stimulate the transcription of *Hsfs* and HSPs accumulation. Thus, CNGC6 was found to promote heat tolerance in *Arabidopsis* seedlings.

Hydrogen peroxide is a plant signaling molecule that plays a vital role in many environmental stress responses. Lots of studies suggest a key role for CNGCs in controlling H_2_O_2_ production (Walker and Berkowitz, 2013; Cui et al., 2020). To elucidate the relationship between H_2_O_2_ and CNGC6 in thermotolerance, we first determined the transcription levels of *AtRbohB* and *AtRbohD* at the seedling stage using the wild-type plants, a T-DNA insertion mutant (*cngc6*; SALK_042207), and a complementation line (COM12; *cngc6 + CNGC6*; Gao et al., 2012). The result showed that no clear difference existed between the expression levels in these seedlings under normal conditions; however, both of their expression levels were stimulated by high temperatures and varied depending on the expression level of *CNGC6* (Supplementary Figure 1), implying that it had a role in the generation of H_2_O_2_. Thus, we examined endogenous H_2_O_2_ accumulations in these seedlings using the special fluorescent probe CM-H_2_DCFDA. This probe can be transported into cells, where its acetate groups are passively cleaved by intracellular esterases, producing the fluorescent compound dichlorodihydrofluorescein (DCF; Chozinski et al., 2016).

**Figure 1:**
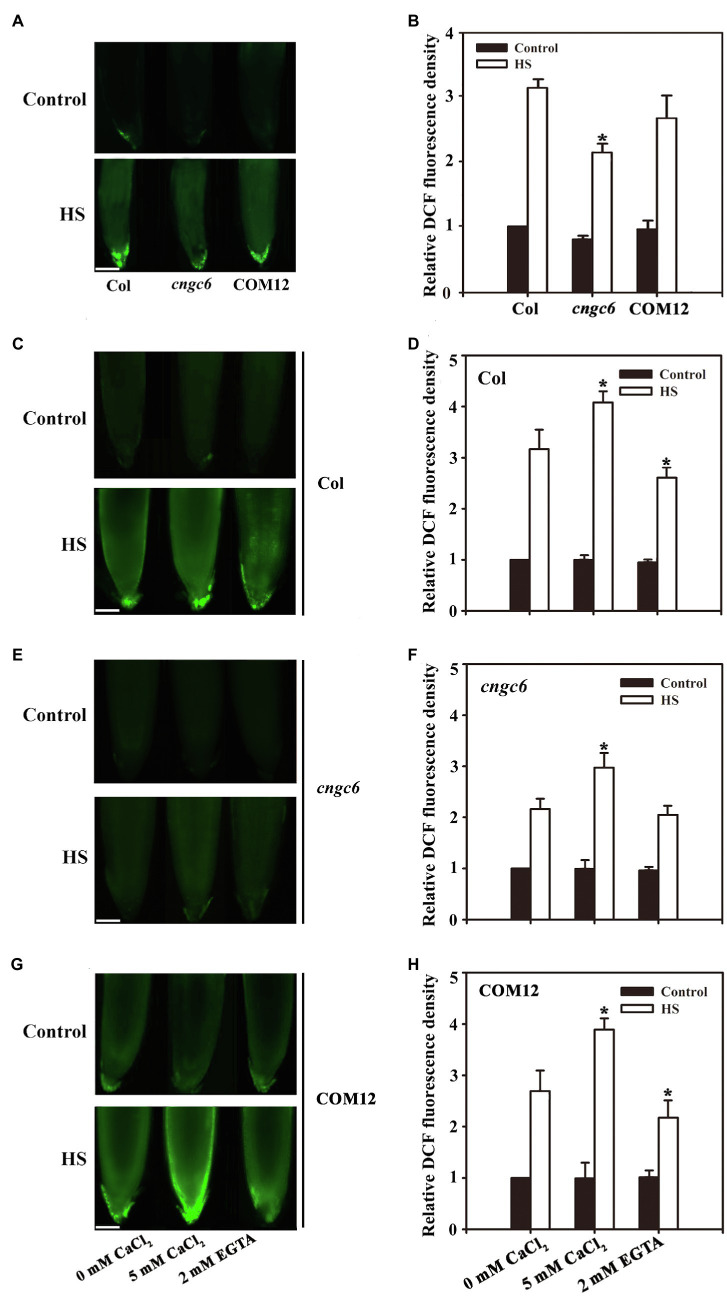
Effects of calcium ions (Ca^2+^) on hydrogen peroxide (H_2_O_2_) accumulation in *Arabidopsis* seedlings. **(A)** About 8-day-old wild-type (WT), *cngc6*, and COM12 seedlings grown at 22°C were exposed to 45°C (heat shock, HS) or maintained at 22°C (Control) for 30 min. The H_2_O_2_ levels in the seedlings were then examined by fluorescence microscopy using roots dyed with 5-(and-6)-chloromethyl-29,79-dichlorodihydrofluorescein diacetate (CM-H_2_DCFDA). Bar = 100 μm. **(B)** Relative dichlorodihydrofluorescein (DCF) fluorescence densities in the roots. The data presented are the means ± SE of measurements taken from five independent experiments with at least 10 roots for each treatment. ^*^p < 0.05 vs. Col (Student’s *t*-test). **(C,E,G)** About 8-day-old seedlings of wild-type **(C)**, *cngc6* **(E)**, and COM2 **(G)** were exposed to 45°C (HS) or maintained at 22°C (Control) for 30 min. The H_2_O_2_ levels in the plants were then examined by fluorescence microscopy using roots stained with CM-H_2_DCFDA. Bar = 100 μm. **(D,F,H)** The relative DCF fluorescence densities in the roots of wild-type **(D)**, *cngc6* **(F)**, and COM2 **(H)**. The data presented are the means ± SE of measurements taken from five independent experiments with at least 10 roots for each treatment. ^*^p < 0.05 vs. 0 mM CaCl_2_ (Student’s *t*-test).

Fluorescence analysis indicated that under normal conditions (22°C), no clear difference in the abundance of H_2_O_2_ was observed among the seedlings. After HS treatment at 45°C for 30 min (Wang et al., 2014), the H_2_O_2_ level increased by 208% in the wild-type seedlings, higher than the increase observed in *cngc6* (108%); however, it was nearly rescued in COM12 seedlings (187%; Figures 1A,B). We also found that not all the production of H_2_O_2_ responsive to HS was inhibited in *ncgc6* mutant. Thus, these results suggest that the production of H_2_O_2_ observed after HS treatment was partially due to the activation of CNGC6.

### Effect of Ca^2+^ on the H_2_O_2_ Accumulation in the Wild-Type Seedlings

Cyclic nucleotide-gated ion channel 6 is a heat-responsive Ca^2+^-permeable channel in the PM of plant cells (Gao et al., 2012). Ca^2+^ is one of the most multifunctional ions existed in eukaryotes, and it has been confirmed to coordinate with H_2_O_2_ in many physiological processes (Ferreira et al., 2003). Thus, it is reasonable to consider that CNGC6 elevates the H_2_O_2_ level through Ca^2+^ to induce thermotolerance.

To test this hypothesis, the H_2_O_2_ levels were examined in the wild-type, *cngc6*, and COM12 seedlings pre-treated with 5 mM CaCl_2_ or 2 mM EGTA (a Ca^2+^ chelator) before germination as described previously (Liu et al., 2005; Peng et al., 2019). Fluorescence analysis showed that under normal growth conditions, the H_2_O_2_ levels in wild-type, *cngc6*, and COM12 seedlings were rather stable. However, under HS conditions, 5 mM Ca^2+^ treatment elevated the H_2_O_2_ level to 411, 303, and 389% of their individual controls in the wild-type, *cngc6*, and COM12 seedlings, respectively. Whereas 2 mM EGTA reduced the increase in H_2_O_2_ to 245 and 213% of the wild-type and COM12 controls, respectively, but there was no clear effect on the *cngc6* mutant (Figures 1C–H).

### Effects of H_2_O_2_ on the Thermotolerance of *cngc6* Seedlings

**Figure 2:**
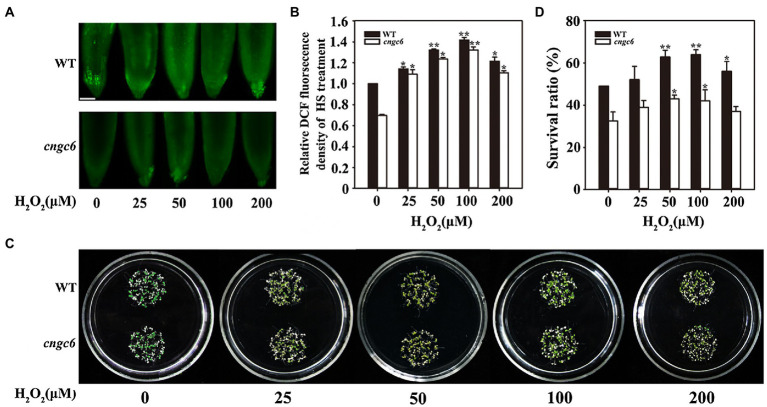
Effects of H_2_O_2_ on the thermotolerance of WT and *cngc6* seedlings. **(A)** About 8-day-old WT and *cngc6* seedlings grown at 22°C were pre-treated with 2 ml of 0, 25, 50, 100, or 200 mM H_2_O_2_ for 8 h and then exposed to 45°C (HS) for 30 min. The H_2_O_2_ levels were then assessed by fluorescence microscopy in roots stained with CM-H2DCFDA. Bar = 100 mm. **(B)** Relative DCF fluorescence densities in the roots. The data presented are means ± SE of measurements taken from at least 10 roots for each treatment. ^*^p < 0.05 and ^**^p < 0.01 vs. 0 mM H_2_O_2_ (Student’s *t*-test). **(C)** Seedlings were exposed to 45°C for 100 min, then returned to 22°C and photographed 5 days later. **(D)** Survival ratios of the seedlings after HS treatment. The data presented are means ± SE of at least five independent experiments with 50 seedlings per experiment. ^*^p < 0.05 vs. 0 mM H_2_O_2_ (Student’s *t*-test).

Subsequently, a solution containing a series of concentrations of H_2_O_2_ was used to pre-treat the wild-type and *cngc6* seedlings. Under HS conditions, the internal H_2_O_2_ level was higher in the wild-type seedlings than in the *cngc6* seedlings. Exogenous application of H_2_O_2_ stimulated the internal H_2_O_2_ level in these seedlings depending on the H_2_O_2_ concentration, reaching a maximum value at 100 μM and decreasing slightly at 200 μM (Figures 2A,B). The survival ratios of the wild-type and *cngc6* seedlings changed in the same manner as their internal H_2_O_2_ levels, reaching the maximum at 100 μM (Figures 2C,D).

Taken together, these results (Figures 1, 2) showed that heat-responsive Ca^2+^ channel CNGC6 regulated H_2_O_2_ production; however, an increased internal H_2_O_2_ level rescued the impaired thermotolerance of the CNGC6-deficient mutant, indicating H_2_O_2_ involvement in CNGC6 signaling as a downstream factor.

### *AtRbohB* and *AtRbohD* Overexpression in a *cngc6* Background Increases Thermotolerance

**Figure 3:**
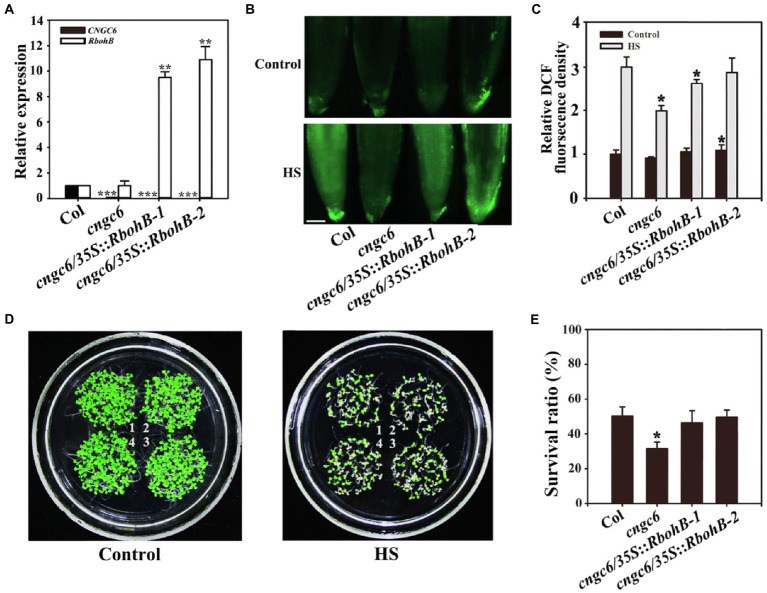
Improved thermotolerance through *AtRbohB* overexpression in a *cngc6* background. **(A)** Real-time quantitative PCR (RT-qPCR) analysis of *AtCNGC6* and *AtRbohB* transcription in wild-type, *cngc6*, *cngc6*/*35S*::*RbohB-1*, and *cngc6*/*35S*::*RbohB-2* plants. The experiments were repeated three times with similar results. Each data point represents the mean ± SD (*n* = 3). Asterisks indicate a significant difference relative to Col (Student’s *t*-test: ^**^p < 0.01 and ^***^p < 0.001). **(B)** About 8-day-old wild-type, *cngc6*, *cngc6*/*35S*::*RbohB-1*, and *cngc6*/*35S*::*RbohB-2* seedlings grown at 22°C were exposed to 45°C (HS) or maintained at 22°C (Control) for 30 min. The H_2_O_2_ levels in the plants were then examined by fluorescence microscopy using roots stained with CM-H_2_DCFDA. Bar = 100 μm. **(C)** The relative DCF fluorescence densities in the roots. The data presented are the means ± SE of measurements taken from five independent experiments with at least 10 roots for each treatment. ^*^p < 0.05 vs. Col. **(D)** Seedlings grown at 22°C were exposed to 45°C (HS) or maintained at 22°C (Control) for 100 min, then returned to 22°C and photographed 5 days later. The clusters are as follows: 1, wild-type; 2, *cngc6*; 3, *cngc6*/*35S*::*RbohB-1*; and 4, *cngc6*/*35S*::*RbohB-2*. **(E)** Survival ratios of the seedlings after HS treatment. The data presented are the means ± SE of at least five independent experiments with 50 seedlings per experiment. ^*^p < 0.05 vs. Col (Student’s *t*-test).

**Figure 4:**
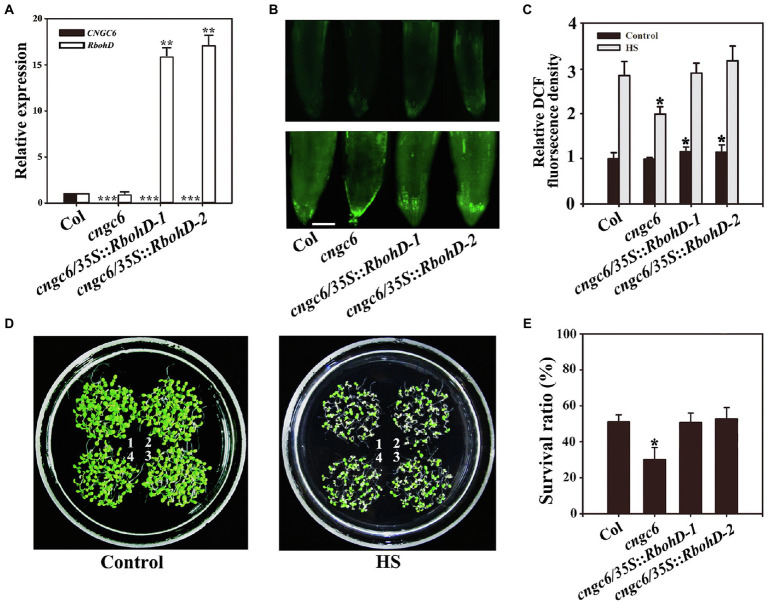
Improved thermotolerance through *AtRbohD* overexpression in a *cngc6* background. **(A)** RT-qPCR analysis of *AtCNGC6* and *AtRbohD* transcription in wild-type, *cngc6*, *cngc6*/*35S*::*RbohD-1*, and *cngc6*/*35S*::*RbohD-2* plants. The experiments were repeated three times with similar results. Each data point represents the mean ± SD (*n* = 3). Asterisks indicate a significant difference relative to Col (Student’s *t*-test: ^**^p < 0.01 and ^***^p < 0.001). **(B)** About 8-day-old wild-type, *cngc6*, *cngc6*/*35S*::*RbohD-1*, and *cngc6*/*35S*::*RbohD-2* seedlings grown at 22°C were exposed to 45°C (HS) or maintained at 22°C (Control) for 30 min. The H_2_O_2_ levels in the plants were then examined by fluorescence microscopy using roots stained with CM-H_2_DCFDA. Bar = 100 μm. **(C)** The relative DCF fluorescence densities in the roots. The data presented are the means ± SE of measurements taken from five independent experiments with at least 10 roots for each treatment. ^*^p < 0.05 vs. Col. **(D)** Seedlings grown at 22°C were exposed to 45°C (HS) or maintained at 22°C (Control) for 100 min, then returned to 22°C and photographed 5 days later. The clusters are as follows: 1, wild-type; 2, *cngc6*; 3, *cngc6*/*35S*::*RbohD-1*; and 4, *cngc6*/*35S*::*RbohD-2*. **(E)** Survival ratios of the seedlings after HS treatment. The data presented are the means ± SE of at least five independent experiments with 50 seedlings per experiment. ^*^p < 0.05 vs. Col (Student’s *t*-test).

We even reported that H_2_O_2_ acts as a signal in heat tolerance using the mutants *rbohB* and *rbohD*, which show poor thermotolerance due to a deficiency in H_2_O_2_ (Wang et al., 2014). To further investigate the effect of CNGC6 on H_2_O_2_ signaling under HS conditions, we obtained two *AtRbohB*-overexpressing transgenic lines, *cngc6/35S::RbohB-1* and *cngc6/35S::RbohB-2*, and two *AtRbohD*-overexpressing transgenic lines, *cngc6/35S::RbohD-1* and *cngc6/35S::RbohD-2*, and examined the influences of excess internal H_2_O_2_ on *CNGC6*-deficient mutants under HS conditions. The increased expression of *AtRbohB* and *AtRbohD* was confirmed according to real-time quantitative PCR (RT-qPCR; Figures 3A, 4A).

Dichlorodihydrofluorescein fluorescence analysis indicated that *AtRbohB* and *AtRbohD* overexpression enhanced the internal H_2_O_2_ levels in these transgenic plants under normal and HS conditions (Figures 3, 4). Under normal conditions, no clear phenotypic difference was observed between *cngc6* mutant and these transgenic lines. However, under high temperature conditions, *AtRbohB* or *AtRbohD* overexpression greatly improved the survival ratio of the transgenic lines in comparison with their background *cngc6* according to their individual transcriptional levels (Figures 3, 4).

These results showed that the overexpression of *AtRbohB* or *AtRbohD* restored heat tolerance in a *CNGC6*-deficient mutant, providing genetic proof for the relationship between CNGC6 and H_2_O_2_ in HS signaling.

### Effects of HS on the Thermotolerance of the *cngc6/rbohB/D* Triple-Mutant Seedlings

**Figure 5:**
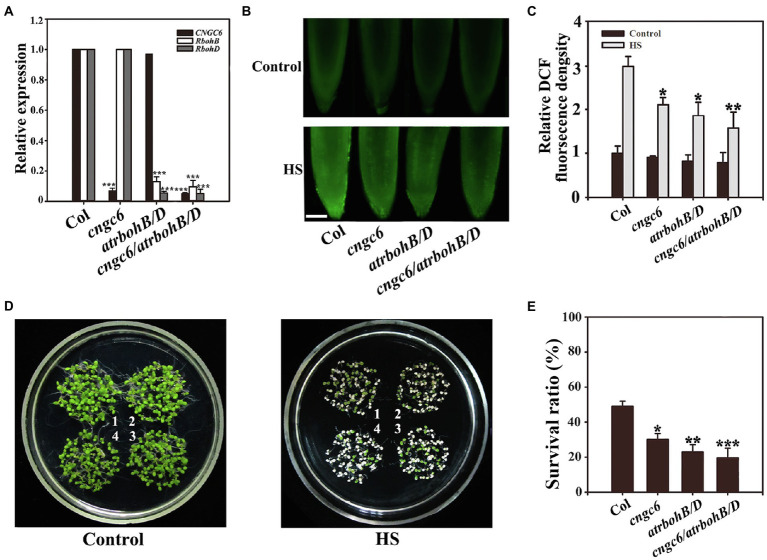
Survival status of the *cngc6/rbohB/D* triple mutant. **(A)** RT-qPCR analysis of cyclic nucleotide-gated ion channel 6 (*CNGC6*), *RbohB* and *RbohD* transcription in wild-type, *cngc6*, *rbohB/D*, and *cngc6/rbohB/D* seedlings. The experiments were repeated three times with similar results. Each data point represents the mean ± SD (*n* = 3). Asterisks indicate a significant difference relative to Col; ^***^p < 0.001 (Student’s *t*-test). **(B)** About 8-day-old wild-type, *cngc6*, *rbohB/D*, and *cngc6/rbohB/D* seedlings grown at 22°C were exposed to 45°C (HS) or maintained at 22°C (Control) for 30 min. The H_2_O_2_ levels in the seedlings were then examined by fluorescence microscopy using roots stained with CM-H_2_DCFDA. Bar = 100 μm. **(C)** Relative DCF fluorescence densities in the roots. The data presented are the means ± SE of measurements taken from five independent experiments with at least 10 roots for each treatment. ^*^p < 0.05, and ^**^p < 0.01 vs. Col (Student’s *t*-test). **(D)** About 8-day-old seedlings grown at 22°C were exposed to 45°C (HS) or maintained at 22°C (Control) for 100 min, then returned to 22°C and photographed 5 days later. The clusters are as follows: 1, wild-type; 2, *cngc6*; 3, *rbohB/D*; and 4, *cngc6/rbohB/D*. **(E)** Survival ratios of the seedlings after HS treatment. The data presented are the means ± SE of at least five independent experiments with 50 seedlings per experiment. ^*^p < 0.05 and ^**^p < 0.01 vs. Col (Student’s *t*-test).

To further examine the roles of CNGC6 and H_2_O_2_ in plant thermotolerance, we obtained the *cngc6/rbohB/D* triple mutant by crossing, which was deficient in *CNGC6*, *RbohB*, and *RbohD* transcription according to RT-qPCR analysis (Figure 5A). Under normal and HS conditions, the H_2_O_2_ level in the *cngc6/rbohB/D* seedlings was similar to that in the *rbohB/D* seedlings (Figures 5B,C), revealing that the deficiency of *CNGC6* did not remarkably reduce H_2_O_2_ accumulation in the *rbohB/D* seedlings. Under normal conditions, *cngc6/rbohB/D* seedlings showed similar phenotypes with other seedlings (Figure 5D, Control). Under HS conditions, the survival ratio of the *cngc6/rbohB/D* seedlings was near to that of the *rbohB/D* seedlings (Figures 5D,E), showing that the deficiency of *CNGC6* did not obviously aggravate the heat susceptibility of *rbohB/D*.

### Effects of H_2_O_2_ on the Activity of Ca^2+^-Permeable Channel

The results provided evidence of the function of CNGC6 on the H_2_O_2_-mediated acquisition of heat tolerance. In *Arabidopsis*, a specific role for H_2_O_2_ in regulating Ca^2+^ mobilization has also been found (Islam et al., 2019).

**Figure 6:**
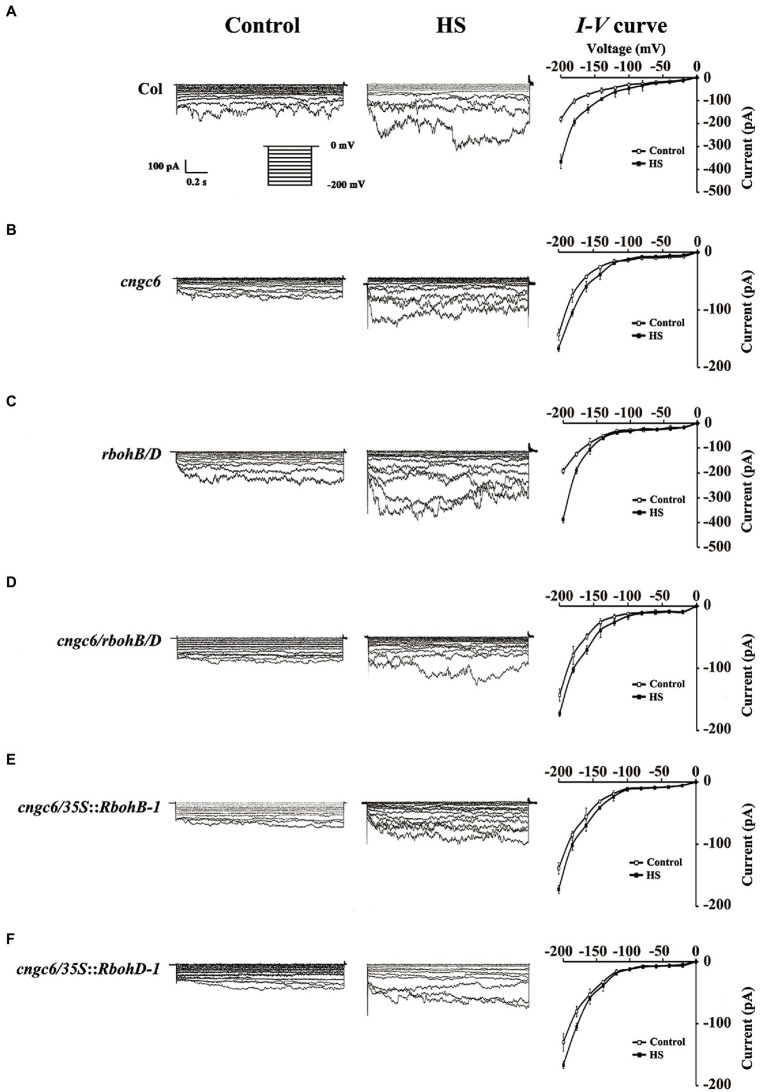
Patch-clamp analysis of Ca^2+^-permeable channels in wild-type, *cngc6*, *rbohB/D*, *cngc6/rbohB/D*, *cngc6*/*35S*::*RbohB-1*, and *cngc6*/*35S*::*RbohD-1* seedlings. The Ca^2+^ current before HS (at 22°C, control) and after HS (at 37°C, HS) was compared in the root cell protoplasts of 8-day-old wild-type **(A)**, *cngc6* **(B)**, *rbohB/D* **(C)**, *cngc6/rbohB/D* **(D)**, *cngc6*/*35S*::*RbohB-1* **(E)**, and *cngc6*/*35S*::*RbohD-1* **(F)** plants. The Ca^2+^ current was recorded by step voltage clamp. Each trace is a representative current from six protoplasts. Currents in the protoplasts are shown in the left and middle columns, respectively. The *I–V* curve is shown in the right column (mean ± SD, *n* = 6).

To confirm whether H_2_O_2_ influences the action of heat-responsive Ca^2+^-permeable channels, we determined the effects of internal H_2_O_2_ on the function of CNCG6 in the PM of root protoplasts of *Arabidopsis* with the whole-cell patch-clamp technique (Gao et al., 2012; Peng et al., 2019). Under normal conditions at 22°C, the Ca^2+^ current in *cngc6* (−136 pA) was lower than in the wild-type (−178 pA) at −200 mV. Under HS at 37°C, the inward Ca^2+^ current was swiftly elevated to −375 pA in the wild-type within 1 min. However, only a slight increase (to −171 pA) was observed in *cngc6* (Figures 6A,B), in accordance with our previous reports (Gao et al., 2012; Peng et al., 2019; Niu et al., 2020). In the *rbohB/D* double mutant with low internal H_2_O_2_ levels, the Ca^2+^ currents exhibited similar changing trends to those in the wild-type under both of normal and HS conditions (Figure 6C). However, in the *cngc6/rbohB/D* triple mutant, the Ca^2+^ currents showed no clear difference with those in *cngc6* under normal and HS conditions (Figure 6D). In two transgenic lines with high endogenous H_2_O_2_ levels, *cngc6/35S::RbohB-1* and *cngc6/35S::RbohD-1*, the Ca^2+^ currents were similar to those of *cngc6* (non-transgenic background; Figures 6E,F), indicating that H_2_O_2_ had no obvious affection on the activity of Ca^2+^ channel.

### Effect of CNGC6 on the Transcription of *Hsf* and the Expression of AtHSP21 and AtHSP17.7 Through H_2_O_2_

**Figure 7:**
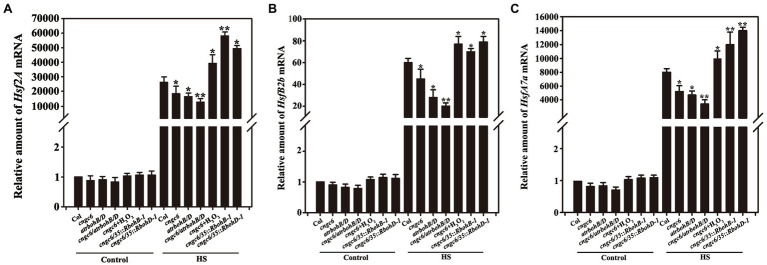
Analysis of the effects of CNGC6 on H_2_O_2_-induced *Hsfs* by RT-qPCR. About 10-day-old seedlings grown at 22°C was exposed to 37°C (HS) or maintained at 22°C (Control) for 60 min, then used for analysis of *Hsf* (**A**, *Hsf2A*; **B**, *HsfA7a*; and **C**, *HsfB2b*) mRNA expression. The data are the mean ± SE of at least five independent experiments. ^*^p < 0.05 and ^**^p < 0.01 vs. Col (Student’s *t*-test).

To investigate the underlying mechanisms of CNGC6- and H_2_O_2_-induced plant thermotolerance, the mRNA level of *Hsf* in the wild-type, *cngc6*, *rbohB/D*, and *cngc6/rbohB/D* seedlings as well as in the two individual *RbohB*- and *RbohD*-overexpressing transgenic lines (*cngc6/35S::RbohB-1* and *cngc6/35S::RbohD-1*) was analyzed using RT-qPCR. Under normal conditions, there was no clear difference among the levels in these seedlings (Figure 7, Control). After the HS treatment, *Hsf* (*Hsf2A*, *HsfA7a*, and *HsfB2b*) mRNA levels were dramatically elevated. However, in *cngc6*, *rbohB/D*, and *cngc6/rbohB/D* seedlings, they were lower than in the wild-type seedlings (and lowest for *cngc6/rbohB/D*) but they were significantly stimulated by 50 μM H_2_O_2_ and were activated in the two transgenic lines compared with their background *cngc6* (Figure 7, HS).

**Figure 8:**
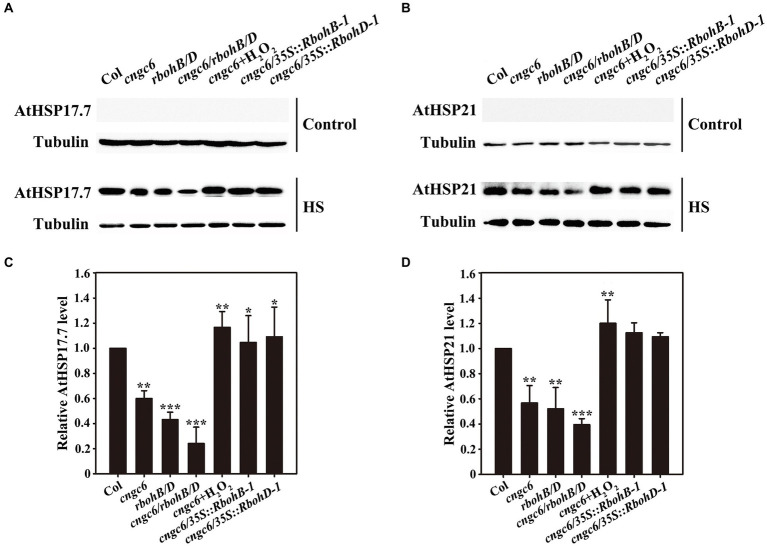
Effects of CNGC6 *via* H_2_O_2_ on AtHSP17.7 and AtHSP21 expression. **(A,B)** About 10-day-old seedlings grown at 22°C were exposed to 37°C (HS) or maintained at 22°C (Control) for 2 h. Total protein was then extracted, separated by SDS-PAGE, and analyzed by Western blotting. Tubulin was used as an internal control. Three independent experiments were carried out; the results indicate similar trends in protein accumulation. **(C,D)** The relative HSP17.7 **(C)** and HSP21 **(D)** level after HS treatment. The data presented are the means ± SE of measurements taken from three independent experiments and represents the relative intensity of each signal. ^*^p < 0.05, ^**^p < 0.01, and ^***^p < 0.001 vs. Col (Student’s *t*-test).

Heat shock proteins, as molecular chaperones, are crucial for all organisms to survive under severe stress through the maintenance of proteostasis (Akerfelt et al., 2010). Thus, we subsequently determined the influences of CNGC6 and H_2_O_2_ on the expression of AtHSP17.7 and AtHSP21 in these plants using Western blotting analysis. Neither AtHSP17.7 nor AtHSP21 was observed at 22°C; however, both of them accumulated at 37°C (Figure 8). The level of protein expression was lower in the mutants than in the wild-type (and lowest for *cngc6/rbohB/D*), and it was greatly elevated by 50 μM H_2_O_2_ in the *cngc6* mutant. In addition, its accumulation was increased in the *cngc6/35S::RbohB-1* and *cngc6/35S::RbohD-1* plants in comparison with the *cngc6* mutant (non-transformed background; Figure 8). In all these experiments, tubulin was adopted to ensure equal sample loading.

These results revealed that the application of H_2_O_2_ and the overexpression of *AtRbohB* or *AtRbohD* prompted HSP expression in a *cngc6* mutant, providing further evidence that CNGC6 acts upstream of H_2_O_2_ in the HS pathway.

## Discussion

### The Relationships Among Ca^2+^, CNGC6, and H_2_O_2_ Accumulation in Plant Thermotolerance in *Arabidopsis* Seedlings

High external temperatures always result in elevated [Ca^2+^]_cyt_ and the accumulation of H_2_O_2_ in plant cells, as they play crucial roles in the response of plant to HS (Liu et al., 2005; Sun and Guo, 2016). However, the relationship between H_2_O_2_ and Ca^2+^ signaling pathways in thermotolerance is unclear. Herein, our work showed that CNGC6, a heat-activated Ca^2+^-permeable channel, stimulates H_2_O_2_ accumulation to regulate the gene expression of *Hsfs* and HSPs accumulation to promote plant heat tolerance.

Hydrogen peroxide, an essential second messenger in a wide variety of biological processes, is stimulated by various factors to counteract exogenous stresses in plants. We previously reported that H_2_O_2_ acts as a signal in the induction of heat tolerance through NO (Wang et al., 2014). NO was even found to be associated with elevating intracellular levels of free Ca^2+^ under HS conditions (Peng et al., 2019). Recently, several studies have focused on the function of Ca^2+^ in initiating H_2_O_2_ accumulation in plants (Ferreira et al., 2003; Zhao et al., 2011). Therefore, we speculated that there should be a close relationship between Ca^2+^ and H_2_O_2_ in HS signaling pathway.

In plants, the CNGC proteins are expressed differentially in numerous tissues (Zelman et al., 2012). Molecular genetic studies have revealed that CNGCs frequently function in numerous biological processes, including plant growth and development, adaptations to increased Ca^2+^ concentration, and plant responses to abiotic and biotic stresses (Gao et al., 2016; Jha et al., 2016). Our prior work has demonstrated that AtCNGC6 is a heat-activated PM Ca^2+^-permeable channel that conducts Ca^2+^ into the cytoplasm to help regulate HS responses. A T-DNA insertion mutant *cncg6* was used for those investigations due to its lower Ca^2+^ current than the wild-type, which is nearly totally restored in the transgenic line COM12 plants after HS treatment (Gao et al., 2012; Peng et al., 2019), indicating that CNGC6 regulates the influx of Ca^2+^ into plant cells. Thus, we used the *cngc6* mutant and the COM12 plants to examine the relationship between H_2_O_2_ and CNGC6 in plant thermotolerance.

The mRNA level of *AtRbohB/D* is stimulated by HS depending on CNGC6 expression levels (Supplementary Figure 1), indicating that CNGC6 regulates H_2_O_2_ accumulation under HS conditions. Thus, we first examined H_2_O_2_ levels using the fluorescent probe CM-H_2_DCFDA. The results showed that high temperatures stimulated H_2_O_2_ accumulation according to their *CNGC6* expression levels in the seedlings (Figures 1A,B), indicating an important role of CNGC6 in the regulation of H_2_O_2_ production in the HS pathway.

Because of the role of CNCG6 in conducting Ca^2+^ into the cytoplasm in HS-treated plants, we determined the effects of Ca^2+^ on H_2_O_2_ accumulations in the wild-type, *cngc6*, and COM12 seedlings. The results showed that Ca^2+^ increased H_2_O_2_ accumulation in the seedlings under high temperature, whereas the Ca^2+^ chelator EGTA clearly reduced H_2_O_2_ accumulations in the wild-type and COM12 seedlings (Figures 1C–H), indicating that CNGC6-mediated free Ca^2+^ is a crucial factor in promoting H_2_O_2_ signaling. Thus, we propose that CNGC6 participates in stimulating internal H_2_O_2_ levels *via* free Ca^2+^ in the HS pathway. However, EGTA had no clear effect on the H_2_O_2_ level in *cngc6* seedlings, which might be due to the smaller increase in free Ca^2+^ under HS exposure (Figures 1E,F).

### Effects of CNGC6 and H_2_O_2_ on Heat Tolerance in *Arabidopsis* Seedlings

To interpret the effects of CNGC6 and H_2_O_2_ on thermotolerance, we determined the effects of H_2_O_2_ on the survival of wild-type and *cngc6* seedlings exposed to HS conditions. Exogenous applications of H_2_O_2_ enhanced the internal H_2_O_2_ levels and the survival ratios of both of HS-treated wild-type and *cngc6* seedlings (Figure 2). The overexpression of two HS-responsive H_2_O_2_ synthesis-related enzymes, *RbohB* and *RbohD*, simultaneously elevated the internal H_2_O_2_ levels and the survival ratios of these transgenic lines, in comparison with their non-transgenic background *cngc6* under HS conditions (Figures 3, 4), respectively, indicating that an increase in internal H_2_O_2_ restored the heat sensitivity of the mutant plants because of the absence of *CNGC6*. We also identified a strange phenomenon in that a high H_2_O_2_ concentration (200 μM) could not produce a high internal H_2_O_2_ level under HS conditions (Figures 2A,B), which is likely due to plant self-protection against oxidative damage as discussed previously (Wang et al., 2014; Wu et al., 2015).

Next, we obtained the triple mutant *cngc6/rbohB/D*, which showed a phenotype similar to that of the *rbohB/D* double mutant under normal and HS conditions (Figure 5), revealing that deficiencies in *CNGC6* and *RbohB/D* do not aggravate the heat susceptibility due to a deficiency in *RbohB/D*.

Collectively, the upon results provide physiological and genetic proof for the existence of a novel HS signaling pathway in which CNGC6 is activated by high temperatures to mediate H_2_O_2_ accumulation to confer plant thermotolerance.

### Effects of H_2_O_2_ on Ca^2+^ Fluxes in the Responses of *Arabidopsis* Seedlings to HS Stress

Hydrogen peroxide is the especially stable one of ROS and regulates plant growth, development, and stress adaptations. It acts through increasing [Ca^2+^]_cyt_ as a second messenger, by the activation of the PM Ca^2+^-permeable influx channels as a primary part of this process (Ordoñez et al., 2014; Richards et al., 2014; Shabala, 2019). However, only few studies have drawn the opposite conclusion that Ca^2+^ influx influences H_2_O_2_ generation. For example, the silencing of two tomato CNGC genes, *SICNGC1* and *SICNGC14*, was reported to strikingly promote both pathogen-induced and flg22-elicited H_2_O_2_, revealing that two *SICNGCs* inhibit ROS production and attenuate non-host resistance and PAMP-triggered immunity (Zhang et al., 2018). Accordingly, we wondered whether H_2_O_2_ stimulates Ca^2+^ influxes to confer thermotolerance.

A marked elevation in Ca^2+^ current was presented in the response to a swift temperature increase from 22 to 37°C in the wild-type. However, the current was clearly inhibited in *cngc6*, *cngc6/rbohB/D*, *cngc6/35S::RbohB-1*, and *cngc6/35S::RbohD-1* plants but not obviously varied in *rbohB/D* plants (Figure 6), showing no great effect of H_2_O_2_ on the activity of Ca^2+^-permeable channel. These results, in combination with those shown in Figures 2–5, proposed that the HS-induced alteration in Ca^2+^ unidirectionally stimulates H_2_O_2_ signaling in plants. A plausible interpretation for these data is that supplementation with H_2_O_2_, a downstream signal molecule, rescued the heat-susceptible phenotype of the *CNGC6*-deficient seedlings (Figures 2–5) but could not elevate the heat-responsive activity of CNGC6 (Figure 6).

### The Mechanism Underlying the Effects of CNGC6 *via* H_2_O_2_ on Thermotolerance

To examine the mechanisms by which CNGC6 influences heat tolerance *via* H_2_O_2_, we determined the effects of CNGC6 and H_2_O_2_ on *Hsf* transcript and HSP expression under HS conditions.

Heat shock factors are known as downstream elements in the HS signaling pathway to regulate heat tolerance by deciding the expression of HSPs as the response to phosphorylation (Kotak et al., 2007). Our current data indicated that a reduction in the level of CNGC6 prohibits the transcript levels of *Hsfs*, whereas applications of H_2_O_2_ and overexpression of *RbohB* and *RbohD* elevates them in *cngc6* plants (Figure 7). Therefore, H_2_O_2_ appears to restore the CNGC6 effects, thereby influencing the *Hsfs* transcription and inducing to thermotolerance.

Heat shock protein genes, stimulated by HSFs linking to promoter elements, are categorized depending on their molecular masses, for example, HSP110, HSP100, HSP90, HSP70, and small HSPs, which are the most important ones among them due to their irreplaceable role in plant tolerance against high temperatures (Carre et al., 2019). To interpret the relationship between CNGC6 and H_2_O_2_ in the HS signaling pathway, we used HSP21 and HSP17.7, two small HSPs, to examine how CNGC6 mediates thermotolerance through H_2_O_2_. Western-blot analysis revealed that the reduced *CNGC6* level in *cngc6* mutant decreased HSP21 and HSP17.7 expression under HS conditions, whereas application of H_2_O_2_ and the overexpression of *RbohB* or *RbohD* in *cngc6* plants increased the accumulation of HSP21 and HSP17.7 (Figure 8), indicating that CNGC6 activated HSP expression *via* H_2_O_2_. Taken together, the mechanism through which CNGC6 influences thermotolerance *via* H_2_O_2_ involves variations in HSP gene expression.

These upon results suggest that CNGC6, the HS-responsive Ca^2+^-permeable channel, takes part in the initiation of HS signaling transduction through H_2_O_2_. We previously suggested a model for the HS signaling pathway in which the HS signal was received by an unknown receptor, resulting in an elevated H_2_O_2_ level and then stimulating NO production and AtCaM3 expression to initiate plant resistance against high temperatures (Xuan et al., 2010; Wang et al., 2014). Additionally, feedback inhibition existed between NO and H_2_O_2_ in the HS signaling pathway in *Arabidopsis* (Wu et al., 2015). AtCaM3 also inhibited excess NO accumulation and enhanced plant thermotolerance through stimulating *S*-nitrosoglutathione reductase by direct binding (Zhang et al., 2020). Recently, we found that CNGC6 through free Ca^2+^ acts upstream of NO in plant response to HS (Peng et al., 2019). In this work, CNGC6 was also proposed to act upstream of H_2_O_2_ through free Ca^2+^ in the HS pathway. Ca^2+^ and AtCaM3 are associated with HSP gene expression in *Arabidopsis* (Zhang et al., 2009). CaM, upon binding to Ca^2+^, attaches to specific targets, increasing their functions as part of a HS-responsive Ca^2+^ signaling pathway, for instance, CaM-binding protein kinase 3 (Liu et al., 2008) and PP7 (Liu et al., 2007). Thus, these findings suggest that interactions exist among Ca^2+^ channels, H_2_O_2_, NO, and the Ca^2+^/CaM-dependent target proteins to participate in regulating HSP expression in the HS pathway.

## Accession Numbers

Sequence data from this article can be found in GenBank/EMBL under the following accession numbers: *AtRbohB* (At1G09090), *AtRbohD* (AT5G47910), *CNGC6* (At2g23980), and *Actin2* (At3g18780).

## Data Availability Statement

The original contributions presented in the study are included in the article/Supplementary Material, further inquiries can be directed to the corresponding author.

## Author Contributions

BL and LZo conceived the project and designed the research. WW and JZ carried out the phenotypic observation, RT-qPCR analysis, *Arabidopsis* transgenic experiments, and Western blot analysis. WW and LA carried out the whole-cell voltage patch-clamping. LZn and DW participated in the data analysis. LZo wrote the article with contributions from all authors and revised and proofread the manuscript. All authors contributed to the article and approved the submitted version.

### Conflict of Interest

The authors declare that the research was conducted in the absence of any commercial or financial relationships that could be construed as a potential conflict of interest.
