# Supplementary material for: Cyclic Nucleotide-Gated Ion Channel 6 Mediates Thermotolerance in Arabidopsis Seedlings by Regulating Hydrogen Peroxide Production via Cytosolic Calcium Ions
Source: Front Plant Sci. 2021 Jul 14;12:708672. doi: 10.3389/fpls.2021.708672 (PMC8317691; doi:10.3389/fpls.2021.708672)
Supplement: Supplementary file 1 [file Table_1.DOC]

**SUPPLEMENTARY MATERIAL**

**Figure S1.** Analysis of the effects of CNGC6 on *AtRbohB* and *AtRbohD* transcription by RT-qPCR.

Eight-day-old wild-type, *cngc6*, and COM12 seedlings grown at 22°C were exposed to 45°C for 0 to 40 min, then used for analysis of *AtRbohB* (A) and *AtRbohD* (B) mRNA expression. The experiments were repeated three times with similar results.

**
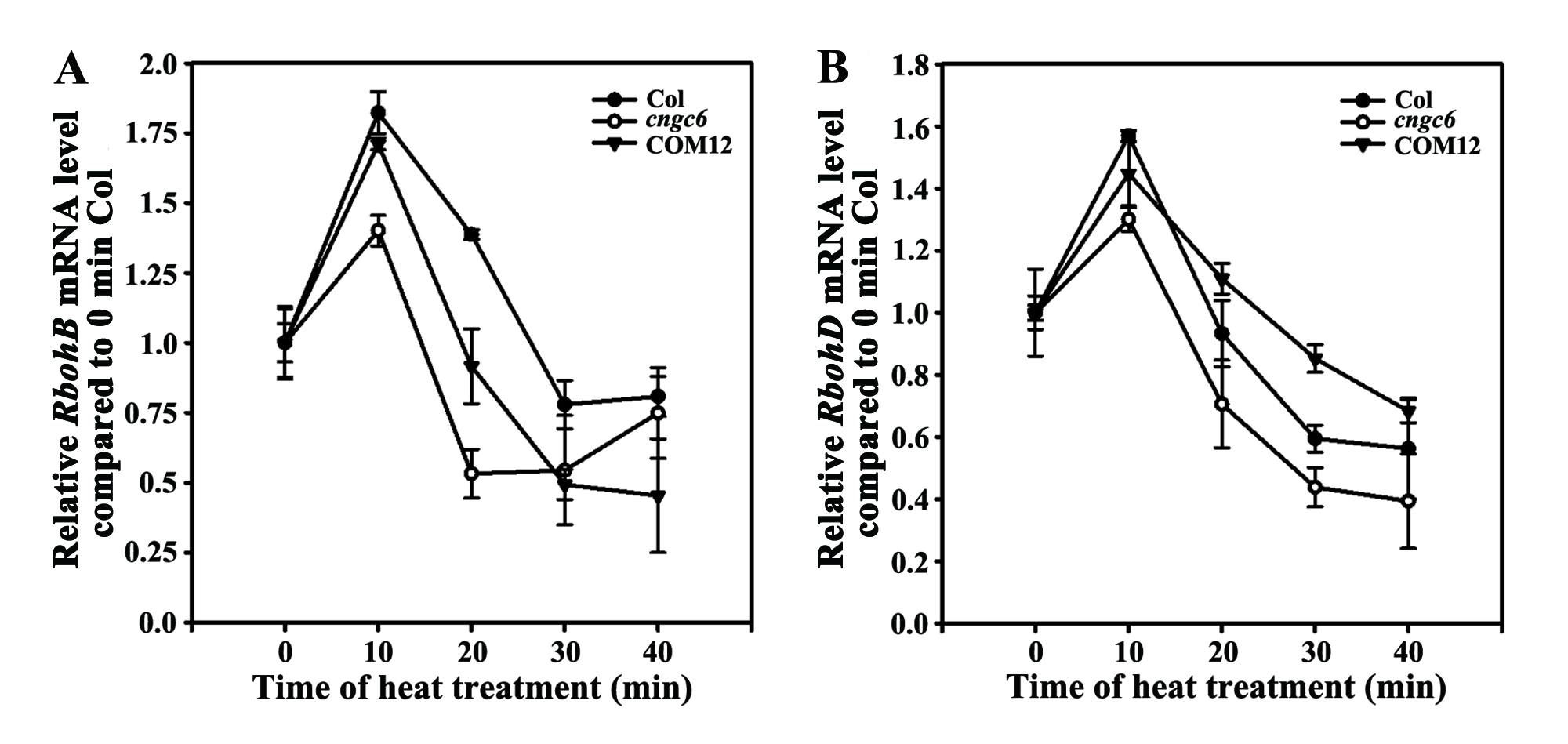
**

**Table S1.** Primers used for real-time quantitative RT-qPCR

| Primer name | Sequence (5'-3') |
| --- | --- |
| RBOHB-F | AATAGGCTTGCACGCTATATCT |
| RBOHB-R | GACGTAGGCCACTAACATAAGA |
| RBOHD-F | ATTACAAGCACCAAACCAG |
| RBOHD-R | TTCTCCGACCATCTCACTA |
| CNGC6-F | CCGCGGAGAGCTTGTTATAG |
| CNGC6-R | CCGGCAGTTCTCTTCAGTTC |
| Actin2-F | GGTAACATTGTGCTCAGTGGTGG |
| Actin2-R | AACGACCTTAATCTTCATGCTGC |
